# Reduced firing rates of high threshold motor units in response to eccentric overload

**DOI:** 10.14814/phy2.13111

**Published:** 2017-01-20

**Authors:** Tom G. Balshaw, Madhu Pahar, Ross Chesham, Lewis J. Macgregor, Angus M. Hunter

**Affiliations:** ^1^ Physiology, Exercise and Nutrition Research GroupUniversity of StirlingStirlingScotlandUnited Kingdom; ^2^ Computing Science and MathematicsUniversity of StirlingStirlingScotlandUnited Kingdom

**Keywords:** Decomposed electromyography, lengthening contractions, MVC

## Abstract

Acute responses of motor units were investigated during submaximal voluntary isometric tasks following eccentric overload (EO) and constant load (CL) knee extension resistance exercise. Ten healthy resistance‐trained participants performed four experimental test sessions separated by 5 days over a 20 day period. Two sessions involved constant load and the other two used eccentric overload. EO and CL used both sessions for different target knee eccentric extension phases; one at 2 sec and the other at 4 sec. Maximal voluntary contractions (MVC) and isometric trapezoid efforts for 10 sec at 70% MVC were completed before and after each intervention and decomposed electromyography was used to measure motor unit firing rate. The firing rate of later recruited, high‐threshold motor units declined following the 2‐sec EO but was maintained following 2sec CL (*P *<* *0.05), whereas MUFR for all motor units were maintained for both loading types following 4‐sec extension phases. MVC and rate of force development where maintained following both EO and CL and 2 and 4 sec phases. This study demonstrates a slower firing rate of high‐threshold motor units following fast eccentric overload while MVC was maintained. This suggests that there was a neuromuscular stimulus without cost to the force‐generating capacity of the knee extensors.

## Introduction

The force capacity of skeletal muscle during eccentric contraction is far greater than that of concentric contraction (Hamill and Knutzen 2009). As such, most conventional resistance exercises using one load for both phases of a dynamic contraction will typically result in the eccentric phase being undertrained (Hortobágyi et al. 2001). This issue has been addressed by using eccentric overload resistance exercise that employs heavier loading during the eccentric phase than in the subsequent concentric phase (Doan et al. 2002) which may (Nichols et al. 1995; Hortobágyi and DeVita 2000; Hortobágyi et al. 2001; Norrbrand et al. 2008; Friedmann‐Bette et al. 2010; Walker et al. 2016) result in greater adaptation. Potential mechanisms responsible for superior adaptation may be: firstly, during eccentric contraction the muscle and its constituent spindles lengthen which will stimulate neural afferent firings to the central nervous system (Enoka 1996) to increase motor unit recruitment to the active muscle (Søgaard et al. 1996; Enoka 1996; Duchateau and Enoka 2016). Potential mechanisms for this occurrence are from reduced intracortical inhibition alongside increased intracortical facilitation (Gruber et al. 2009; Howatson et al. 2011). Relative to concentric contractions, reduced inhibition will likely occur from blunted GABAergic inhibitory interneuron activity (Howatson et al. 2011), whereas increased facilitation is from involvement of a larger brain area (Gruber et al. 2009). This occurrence is likely to stimulate more sarcoplasmic calcium release giving a greater stimulus for myocellular adaptation (Gehlert et al. 2015). Secondly, eccentric overload (EO) has previously been shown to increase the cross‐sectional area of type IIX, but not other muscle fiber types in resistance‐trained individuals (Friedmann‐Bette et al. 2010).

In untrained populations, eccentric adaptation is predominantly driven by muscle damage incurred (Higbie et al. 1996; Farthing and Chilibeck 2003; Shepstone et al. 2005). However, in resistance‐trained individuals, muscle damage is likely to be far less as they are more accustomed to lengthening contractions (Clarkson and Hubal 2002). Recently, it was demonstrated (Walker et al. 2016) in resistance‐trained individuals that superior strength adaptation following EO training probably occurred through neural rather than hypertrophic mechanisms following the second 5 week mesocycle.

Given that increased neural stimulus along with increased type IIX are responsible for superior strength adaptation in trained individuals; it is possible that the larger motor units, or at least (according to Henneman's size principle: Henneman 1957), the later recruited higher threshold motor units may be affected during EO to stimulate such adaptation. Therefore, it is likely that these responses will result in altered motor neuron firing of high threshold motor units which can be measured by decomposed electromyography (dEMG). This technology enables the measurement of a sample of recruited motor units (~40) representative of the active motor unit population (McManus et al. 2015), along with the action potential discharge rate from each motor unit onto the sarcolemma; this can be recorded during voluntary contraction at any given load. To calculate motor unit firing rate (MUFR), it is necessary to decompose the recorded action potentials using algorithms as described previously (De Luca and Contessa 2012).

It is also important to consider that EO resistance exercise performed at fast velocities results in greater strength gains than equivalent training completed at a slower velocity (Paddon‐Jones et al. 2001; Farthing and Chilibeck 2003). As these studies performed maximal contractions the forces exerted were higher due to the inverse relationship between force and velocity for resistance exercise eccentric contractions (Farthing and Chilibeck 2003). This greater strength adaptation, from fast velocity eccentric contractions, occurs from increases in type IIX muscle fibers (Paddon‐Jones et al. 2001) suggesting that their corresponding motor units were stimulated during the contractions. Therefore, EO and constant load (CL) exercises with varied eccentric phase velocities place different demands onto the muscle resulting in altered acute neural responses (Carroll et al. 2001).

To date, we have widespread understanding of MUFR responses during CL but not in EO exercise; investigation of these responses is crucial in our understanding of neural mechanisms at high voluntary force levels at different velocities while controlling for the effects of force. This will enable further research on chronic responses to varying modifications of EO in different cohorts, such as elderly and clinical populations. Given eccentric training has improved muscle strength and reduced risk of falling in the elderly (Isner‐Horobeti et al. 2013), this is an important area of research. By using dEMG technology researchers will be able to accurately monitor neural response changes to EO, over time, in relation to increases in strength. This will be important given the anabolic resistance that many of these populations suffer from. Accordingly, we propose to establish the effects of EO in young, healthy, active males without fatigue which may mask the effects of EO. Recording dEMG during voluntary contraction before and after either EO or CL contractions, performed at fast and slow velocities, will establish this. From this, following EO, we would expect to observe a greater decline in the later recruited high threshold motor units, largely representative of type IIb/X. Furthermore, it would be expected that these changes would be more pronounced in the faster velocities compared to the slower ones. Therefore, the primary aim of this study was to determine MUFR characteristics following EO. Specifically, this will enable us to establish the impact of EO on MUFR by comparing early versus later recruited motor units. The secondary aim was to establish the impact of eccentric contraction velocity on the above.

## Materials and Methods

### Participants

Ten healthy, active males, who had been involved in weekly resistance training for at least 1 year (aged: 22.2 ± 1.3 years, body mass: 78.4 ± 6.1 kg, height: 1.80 ± 0.06 m) took part in the study once they had provided written informed consent. Ten participants were chosen based on repeated measures cross over design using MUFR effect size (pre postchange from fast EO) pilot data at 90% power using G*Power 3.1 priori sample size analysis. Local ethical approval was granted before participant recruitment commenced. The principles of the Declaration of Helsinki (World Medical Association 2013) were followed throughout the study. Participants refrained from exhaustive exercise in the 24‐h before each test session. In addition, food and fluid intake diaries were recorded for 48‐h before the first visit to the laboratory and before each experimental testing session.

### Concentric knee extension 3RM

Unilateral concentric strength of the knee extensor musculature of each participant's dominant leg was conducted on a dynamometer (Biodex 3 dynamometer, Biodex Medical Systems, Shirley, NY). Concentric knee extension 3RMs were performed in isotonic mode after accounting for the effects of gravity; participants were required to exceed the programmed level of torque before movement of the axis attachment would occur (Remaud et al. 2005). Additional knee extensor torque was absorbed by the dynamometer and returned as increases in angular velocity (Kovaleski et al. 1995). Thus, load was held constant and velocity varied subject to the torque produced by the knee extensor musculature (Remaud et al. 2005; Power et al. 2010). Three incremental load warm‐up sets (set 1: 10 repetitions, set 2: 5 repetitions, and set 3: 3 repetitions) were performed to prepare participants for 3RM attempts. Extraneous bodily movement during 3RMs was avoided by securing the participants to the dynamometer chair with straps placed across the shoulders, waist, and uninvolved leg. Dynamometer settings were adjusted to align the lateral femoral epicondyle with the dynamometer axis and secure the participant's dominant leg to the axis attachment arm above the lateral malleolus. Dynamometer settings were recorded for each participant to standardize testing in each of their subsequent experimental conditions.

### Experimental protocol

Participants attended the laboratory on seven occasions; three familiarization sessions to adapt the knee extensors and prevent EIMD and four experimental conditions. Following familiarization, one experimental condition was completed, in randomized order, on each of the final four visits. The experimental conditions involved the completion of either CL or EO knee extension efforts in random order. Experimental sessions were conducted at the same time of day to minimize the influence of diurnal variation and each experimental session was separated by at least 5 days.

**Figure 1:**
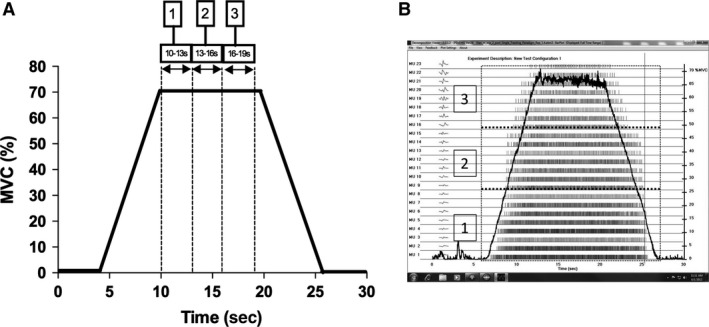
(A) Concentric knee extension isometric trapezoid force trace (MVC%) with 3 plateau phase time periods in which motor unit firing (MUFR) analysis was conducted. (B) Firing rate bar plot. Vertical lines represent the firings of each motor unit and the black line indicates the force trace. The red broken line boxes denote the three identified motor unit populations used for analysis; (1) early‐recruited; (2) mid‐recruited; and (3) later‐recruited motor units. MUFT, motor unit firing rate.

Each experimental test day began with the assessment of unilateral knee concentric extension (three repetition maximum; 3RM). The familiarization during the first three laboratory visits allowed 3RMs to be established within two or three attempts on experimental days and prevented the assessment affecting participant effort during the experimental condition repetitions. A single knee extension isometric maximal voluntary contraction (MVC) was completed 20‐min after the 3RM concentric knee extension efforts, followed by a single submaximal isometric trapezoid effort (Fig. 1A). A 60‐sec recovery period separated the MVC and isometric trapezoid effort to avoid losing the residual effect of the intervention. Four minutes after the isometric trapezoid effort, the first set of concentric knee extension efforts was started.

The experimental condition sets were performed with the same dynamometer configuration and mode as for the 3RM assessment. They consisted of three sets of three unilateral knee concentric and eccentric repetitions with the dominant leg; 3‐min recovery periods were employed between sets to facilitate recovery and avoid accumulative fatigue. The experimental conditions consisted of two EO and two CL conditions, one condition within each loading condition was with a target 2‐sec eccentric knee extension phase, whereas the other was with a 4‐sec eccentric knee extension phase, creating four conditions of CL‐2s, EO‐2s, CL‐4s, and EO‐4s. The CL interventions involved loading of 85% of concentric 3RM in both eccentric and concentric knee extension phases. EO interventions involved loading of 120% of concentric 3RM in the eccentric phase and 85% in the concentric phase. Participants performed the eccentric phase by attempting to match a verbal stop‐watch count (of either 2‐sec or 4‐sec) given by a member of the research team for each repetition. The concentric knee extension phase was performed as explosively as possible to determine if the lengthening during the eccentric phase resulted in increased concentric power. Participants were instructed to transition as quickly as possible between eccentric and concentric phases and to kick out as explosively as possible for each concentric repetition to establish any effects from the stretch shortening cycle. Knee extension repetitions were performed through a minimum 70° range of movement, from 90° of knee flexion to 20° of flexion (0° equaling full extension). A minimum 70° range of motion was used given the large decreases in concentric knee extension force production beyond this range (Knapik et al., 1983). At 9 min after the final knee extension set, participants completed another concentric single knee extension MVC followed 60 sec later by a final isometric trapezoid effort. Work done (J) and time under tension was quantified by the Biodex 3 dynamometer for both concentric and eccentric phases in each condition (Table 1 ). Knee extension kinematic variables (average power and peak velocity) were recorded by software integrated with the Biodex 3 dynamometer and stored electronically for later analysis (Table 2).

**Table 1:** Mean linear slope coefficients (PPS^a^/%MVC) and *y*‐intercepts (PPS) relationships between average firing rate and recruitment threshold of vastus lateralis motor units before (PRE) and after conventional load (CL) and eccentric overload (EO) interventions at 2 and 4 sec contraction velocities

	CL		EO	
	Pre	Post	Pre	Post
Number of motor units 2 sec	18.7 ± 6	17.1 ± 5.3	20.1 ± 5.2	21.6 ± 8
Number of motor units 4 sec	20.6 ± 8.1	18 ± 6.6	19 ± 6.6	17 ± 5.7
Slope coefficient 2 sec	−0.6 ± 0.5	−0.57 ± 0.3	−0.62 ± 0.3	−0.78 ± 0.5
Slope coefficient 4 sec	−0.79 ± 0.3	−0.76 ± 0.4	−0.69 ± 0.3	−0.94 ± 0.7
*Y*−intercept 2 sec	28.5 ± 15.8	25.9 ± 10.9	30.1 ± 8.2	33.5 ± 15.6
*Y*‐intercept 4 sec	34.2 ± 12.5	32.2 ± 11.9	31.4 ± 13.4	36 ± 20.9

a PPS,pulse per second; MVC, Maximal voluntary contractions.

**Table 2:** Total work done and mean time under tension (TUT) for eccentric and concentric phases for Constant Load (CL) and Eccentric Overload (EO) for 2 and 4 sec velocities

	CL		EO	
	Eccentric	Concentric	Eccentric	Concentric
Work done (J) 2 sec	1,765.9 ± 308.8	1,671.8 ± 217.5	2,285.1 ± 534.4^a^	1,659.5 ± 252.6
Work done (J) 4 sec	2,151.5 ± 348.2	1,769.5 ± 263.3	2,667.0 ± 344.3^a^	1,708.1 ± 207.8
TUT (s) 2 sec	16.01 ± 2.4^b^	4.43 ± 1.3	15.4 ± 2.1^b^	5.2 ± 2.4
TUT (s) 4 sec	29.9 ± 2.7	6.4 ± 4.2	30.1 ± 3.6	6 ± 2.4

a *P *<* *0.001 CL vs. EO.

b *P *<* *0.001 fast vs. slow.

### MVC

Isometric knee extension MVCs of 5‐sec duration were also performed on the Biodex dynamometer in the same configuration as the 3RM. The isometric setting was employed during MVCs to assess peak force of the dominant leg knee extensor musculature at a 70° (full extension equaling 0°) joint angle. Before MVCs, participants were instructed to produce the greatest force they could as quickly as possible from the signal to start each MVC. Intense verbal encouragement was provided during all MVCs. Torque data from the dynamometer were recorded by integrated hardware (Bagnoli 16‐channel EMG system, Delsys, Boston) and software (EMGworks^®^ 4.0 Acquisition software, Delsys, Boston). The isometric rate of torque development (RTD) was calculated from the MVC as the average slope of the torque profile from 0 to 50, 0 to 100, 0 to 200, and 0 to 300 msec (Aagaard et al. 2002). The onset of muscle contraction was defined as the point at which the torque curve exceeded the baseline level by 5% of MVC (Blackburn et al. 2009). Baseline resting torque was computed by taking the average reading over 0.5 sec, starting 1‐sec before the onset of muscle contraction for MVC.

### Isometric trapezoid efforts

Isometric concentric knee extension trapezoid efforts used the same set up as for the 3RM and MVCs conducted on the Biodex dynamometer with the participant's dominant leg. Isometric trapezoid efforts involved a 3‐sec quiescent period, a linear 7‐sec ramp‐up in force from 0% to 70% of before‐intervention peak MVC force, 10‐sec holding force levels constant at 70% of peak MVC force, a linear 7‐s ramp‐down from 70% to 0% of MVC peak force, and a final 3‐s quiescent period (Fig. 1A). This contraction provided a stationary signal, long enough for reliable decomposition of sEMG and at the maximal force intensity that could be achieved by all participants. Participants met the required trapezoid force trace as closely as possible via visual feedback displayed on a computer screen positioned at eye level. A 70% of peak MVC target force was selected for the plateau phase as most studies conducting cross‐correlation analysis of single motor units have employed lower target forces (<30% of MVC; Fling et al. 2009). Therefore, findings have been limited to motor units recruited at these lower forces. As the EO and CL interventions involved large forces, it was critical to investigate MUFR responses at as high an isometric target force as possible that could be maintained for the duration of the 10‐sec plateau phase. The selection of greater isometric force during the isometric trapezoid effort permitted the effect of the EO and CL interventions on a larger range of motor units to be assessed.

### Decomposed electromyography

Vastus lateralis (VL) surface EMG was measured and amplified during the isometric trapezoid efforts using a modified Bagnoli 16‐channel EMG system (Delsys, Boston). A five‐pin sensor was provisionally applied to the VL site as recommended by SENIAM for VL bipolar surface electrode configuration (Hermens et al. 2000). The sensor consisted of five cylindrical blunted probes, each with a diameter of 0.5 mm. The probes occupied the four corners and the center of a 5 × 5 mm square. The sensor was pressed forcefully in to the skin while avoiding piercing of the skin and was secured with micropore tape. Before placing and securing the electrode the skin overlying the VL was shaved, cleansed, and abraded. A 5.08 cm diameter reference electrode (HE‐R, Dermatrode, American Imex, Irvine) was applied to the patella of the involved leg. The dEMG system recorded four separate bipolar EMG signals from the 5‐pin sensor probe array at a sampling frequency of 20 kHz. The four signals from each isometric trapezoid effort were filtered with a band width of 20 –1750 Hz (De Luca and Contessa 2012; Hu et al. 2014).

Surface EMG collected during the isometric trapezoid was decomposed using the Precision Decomposition (PD) III algorithms which have previously been extensively detailed elsewhere (De Luca et al. 2006; Nawab et al. 2010) along with its reliability and validity to motor unit firing behavior (De Luca and Hostage 2010; Hu et al. 2013a,b,c, 2014). These algorithms were implemented via EMGworks^®^ 4.0 Analysis software (Delsys, Boston). The PD III algorithms employ the artificial intelligence framework known as “Integrated Processing and Understanding of Signals” to separate the action potentials of different motor units from the overall surface EMG signal. The PD III algorithms are designed to overcome the following issues that are encountered when identifying motor unit action potentials from surface EMG when many motor units are active during a contraction, these include: (1) superposition of motor unit action potentials from different units; (2) the range of amplitudes of action potentials from different motor units; (3) changes in shape of action potentials from detected motor units; 4) similarity of shape of action potentials of different motor units; 5) the interaction of two or more of the factors listed (De Luca et al. 2006).

**Figure 2:**
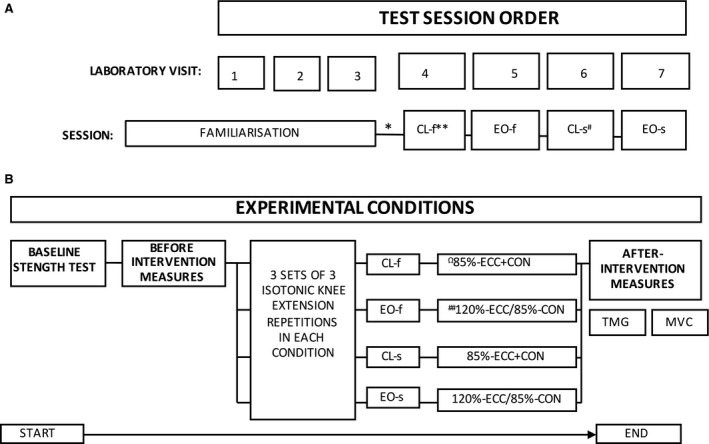
Overall testing session order for (A) and the protocol for an individual experimental test day (B). *denotes randomization of experimental conditions. ** and # denote performance of fast (f) or slow (s) eccentric phases, respectively. Ω85%‐ECC+CON denotes percentage of 3RM lifted in both the eccentric and concentric phases. ^##^120%‐ECC/85%‐CON denotes percentage of 3RM lifted in the eccentric and concentric phases, respectively. The two interventions were CL, constant load; EO, eccentric overload.

Absolute MUFRs (pulses per second) were established for each identified portion of the plateau phase of the isometric trapezoid force trace (Fig. 1A) by extracting data produced from decomposition algorithms with Matlab software (Mathworks, Inc., Natick). The firing rate of motor units from the decomposed surface EMG signals were analyzed by dividing the motor units into tertiles: (1) early‐recruited, (2) mid‐recruited and; (3) later‐recruited motor units (Fig. 1B). This allowed the analysis of three populations which were expected to display differential firing rate characteristics (Eccles et al. 1958). This is a similar concept to that employed by Defreitas et al. (2014) and Carpentier et al. (2001) who defined low and high threshold motor units by comparing <30% and 25% MVC with >25% and 30% MVC, respectively. Whereas our tertile method provides a more objective method of separating the early versus the later recruited motor units available from the sample pool, as opposed to a given amount of force which will be highly variable between participants. Also, the tertile analysis reduces the chance of mid‐recruited motor units from being selected into the wrong category. Specific 3‐s time periods during the isometric trapezoid effort, to establish the most reliable portion of the plateau, were analyzed by calculating average firing rates within each of the three motor unit groups to provide details of each population during different stages of the plateau phase (Fig. 2A). The constant force period of the isometric trapezoid was chosen for analysis to avoid MUFR being influenced by the more complex recruitment and derecruitment phases. These two periods require precise increments and decrements in force compared to the plateau period which simply requires the magnitude of force to be maintained.

The accuracy of the decomposition for each isometric trapezoid effort conducted was assessed with “reconstruct and test” analysis (Nawab et al. 2010; De Luca and Contessa 2012). This is currently considered the most suitable way of validating the decomposition of the surface EMG signal (De Luca et al. 2015). This analysis (Accuracy = 1‐*N*_error_/*N*_truth_ (Where *N*_error_ is the total number of unmatched events, and *N*_truth_ is the total number of true events) assesses the level of firing rate accuracy of each detected motor unit and the number of errors per second, across the entire duration of the isometric trapezoid effort. Each detected motor unit was required to display an accuracy of >85.0% across the entire isometric trapezoid effort to be included for analysis (Stock et al. 2012). Interday reliability of three different time phases (10–13 sec, 13–16 sec, and 16–19 sec) for the plateau section were then assessed with the best one used for subsequent analysis. The recruitment threshold for each motor unit was calculated as the relative force (%MVC) compared with the initial firing as previously described (Ye et al. 2015). Given the accuracy of the motor unit firing is substantially less at the contraction onset in comparison to the plateau phase we only included motor units that were decomposed with >90% accuracy.

### Global EMG

VL EMG was recorded from the participant's dominant leg (Biopac MP100, Biopac Systems Inc, CA) with a bipolar electrode configuration (VERMED A10005‐60 performance plus ECG diagnostic electrodes, Vermont) following shaving, cleansing and abrading the skin recording area. A reference electrode was placed on the lateral malleolus of the participant's dominant leg and secured with micropore tape. EMG was sampled at 2000 Hz and anti‐aliased with a 500 Hz low pass filter. The Biopac MP100 system had an input impedance of 2 MΩ and common mode rejection ratio of >110 Db. From this, sEMG amplitude was determined by transferring the raw signal to root mean square (RMS), which via bespoke sEMG amplifiers (Biopac Systems Inc.). RMS was processed from the sEMG amplitude, using a 100‐msec moving window and averaged at 10 Hz. RMS processing was conducted across the entire waveform for each experimental condition. EMG processing was completed with the software program AcqKnowledge^®^ (Version 3.9, Biopac Systems Inc, CA) according to manufacturer guidelines (Acqknowledge^®^ software guide, 2008).

Once processed, EMG was extracted from experimental condition sets. Eccentric and concentric knee extension phase EMG during experimental condition repetitions was extracted based on synchronized dynamometer axis position data, indicating the start and end of each phase. A voltage channel from the Biodex 3 dynamometer quantifying axis position was calibrated, extracted and recorded during experimental condition knee extension sets with integrated AcqKnowledge^®^ software. Mean EMG from both the eccentric and concentric knee extension phases of the experimental condition sets was normalized to mean EMG from the corresponding muscle action phase recorded during the heaviest successful 3RM attempt at the beginning of the respective test session. Experimental condition EMG was normalized to the dynamic 3RM based on research and findings advocating the use of dynamic normalization methods when investigating dynamic tasks (Albertus‐Kajee et al. 2010; Balshaw and Hunter 2012).

### Statistical analysis

Minitab 16 statistical software (Minitab Ltd., Coventry) was used for all statistical analyses. Normality of force data and HDEMG variables was assessed via Q‐Q plots and constant variance. Repeated measures ANOVAs (4 conditions × 2 time points) were completed to assess differences in firing rate, the maximum number of detected motor units and cross‐correlation coefficients between conditions. A significance level of *P *<* *0.05 was selected to determine statistical differences. Tukey post hoc analysis was used to determine where differences occurred between loading conditions. Where appropriate, 95% lower and upper confidence intervals (CI) and Cohen's *d* effect sizes (ES) calculated by: Cohen's *d *= *Mean*_1_‐*Mean*_2_/SD_pooled_, where SD_pooled_ = √[(SD_1_^2^+ SD_2_^2^)/2]. ES were then interpreted as ≤0.2 = trivial, 0.2–0.5 = small, 0.5–0.8 =  moderate, ≥0.8 = large.

Absolute and relative reliability, as well as interindividual variability in firing rate data were calculated for each motor unit population (early‐, mid‐, and late‐recruited motor units) during the identified time periods (Fig. 2A) of before‐intervention isometric trapezoid efforts on each experimental test day. The reliability of MUFR during the 3 sec time periods was assessed during before‐intervention isometric trapezoid efforts on each experimental test day to assess the suitability of using each section of the isometric trapezoid effort for analysis. Absolute reliability of MUFR data was assessed via intraparticipant CV%. Relative reliability of MUFR data was assessed using ICC. ICC values and 95% CIs were calculated with statistical spread sheets downloaded from www.sportsci.org. Interparticipant variability in MUFR data were assessed using interparticipant CV% to determine if “common” firing rates existed between participants.

## Results

**Figure 3:**
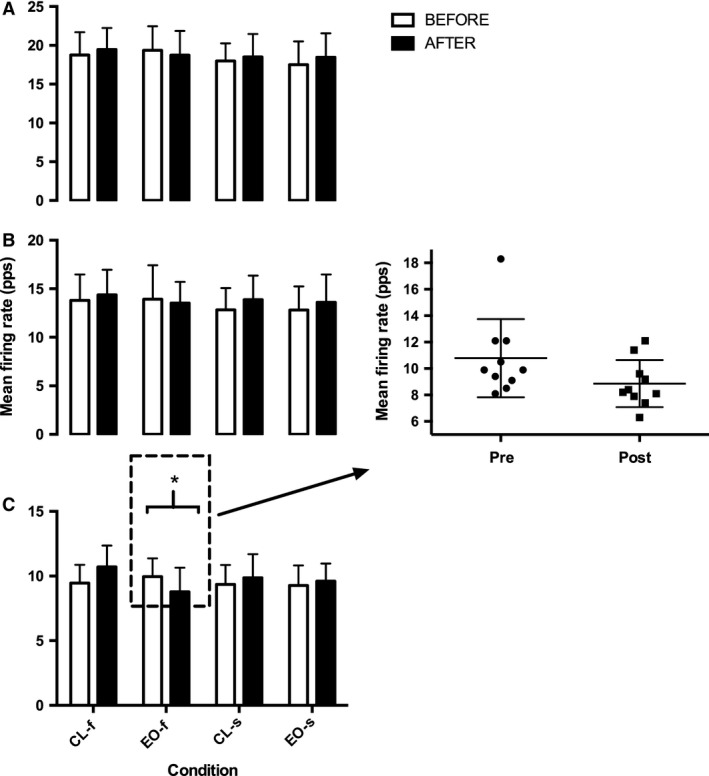
Mean vastus lateralis firing rate (pulses per second, pps) of the constant force phase of the isometric TRAP effort for: (A) early‐; (B) mid‐; and (C) later‐recruited motor units during EO and constant load (CL) conditions with target 2sec (f) and 4sec (s) knee extension eccentric phases. *P =* *0.04 decrease between before‐ and after‐intervention measures. Insert graph is showing individual responses for later recruited motor units before and after EO. EO, eccentric overload.

MUFR of the high threshold motor units (tertile 3) demonstrated a significant interaction effect (*F*_3,27 _= 6, *P *=* *0.002) between the four conditions (CL and EO for 2 sec and 4 sec) and the two time points (pre and post) (Fig. 3C). This was mainly due to the 18% (*P *=* *0.04 ES: 0.79 CI: −3.7 to −0.15) decrease in high‐threshold motor units (tertile 3) MUFR following the 2‐sec EO contraction (Fig. 3C); all participants showed a decline in MUFR except one who maintained MUFR. No changes in MUFR were observed for the low‐ and moderate‐threshold motor units (tertiles 1 and 2; Fig. 3A and B) for either 2‐sec or 4‐sec contractions. Neither were there any significant differences observed for MUFR in any of the other conditions in any of the separate MU populations. When examining the relationship between average MUFR and the recruitment threshold, no differences existed following any of the interventions (Table 1).

**Figure 4:**
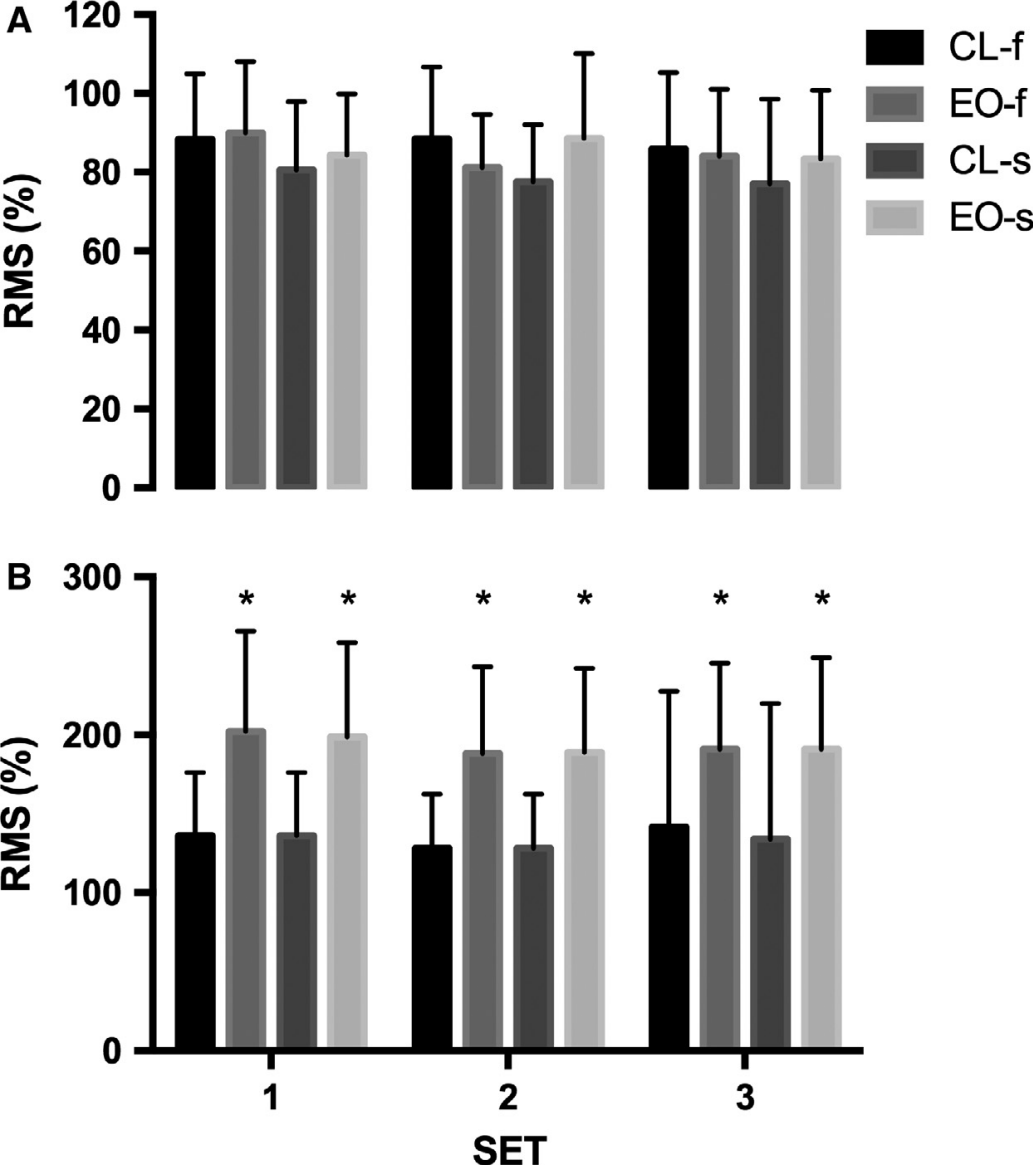
Mean electromyography amplitude of the vastus lateralis during fast (f) and slow (s) CL and EO for: (A) concentric and (B) eccentric phases. *P =* *000.4 CL versus EO across all three sets. EO, Eccentric Overload; CL, Constant load.

No differences in maximum number of detected motor units, MVC and RTD were observed between conditions, velocities, and across time (Table 3). During all contractions the eccentric phase displayed higher RMS than the concentric phase (*P *=* *0.0004; Fig. 4). No within‐condition differences in RMS were shown between 2‐sec and 4‐sec contractions for both phases. Total work done was significantly (*P *=* *0.0006) greater during EO than during CL contractions at both speeds during the eccentric phase (Table 2). Velocity and power during the explosive concentric phase showed no significant differences between the EO and CL contractions for both velocities (Table 4).

**Table 3:** Number of detected motor units for tertile analysis, maximal voluntary contraction (MVC) rate of MVC torque development (RTD) (0–50,0–100, 0–200 & 0–300 msec) measured before and after constant load (CL) Control and eccentric overload (EO) for 2 and 4 sec contractions

	CL		EO	
	Pre	Post	Pre	Post
Motor unit number 2 sec	26.8 ± 6.7	26.4 ± 5.1	25.7 ± 4.5	27.8 ± 7.5
Motor unit number 4 sec	29.7 ± 6.7	24.6 ± 5.4	25.6 ± 6.0	27.6 ± 6.3
MVC (Nm) 2 sec	321.7 ± 41.2	301.5 ± 38.5	332.2 ± 39.1	324.3 ± 39.0
MVC (Nm) 4 sec	327.9 ± 36.0	316.9 ± 41.0	324.0 ± 39.9	313.2 ± 41.0
RTD 0–50 msec (Nm sec^−1^) 2 sec	1811 ± 306	1672 ± 398	1677 ± 357	1751 ± 426
RTD 0–100 msec (Nm sec^−1^) 2 sec	1378 ± 207	1211 ± 209	1276 ± 314	1256 ± 287
RTD 0–200 msec (Nm sec^−1^) 2 sec	1877 ± 325	1785 ± 292	1872 ± 334	1812 ± 317
RTD 0–300 msec (Nm sec^−1^) 2 sec	2030 ± 349	1984 ± 302	2104 ± 343	2081 ± 436
RTD 0–50 msec (Nm sec^−1^) 4sec	1781 ± 375	1730 ± 480	1668 ± 500	1717 ± 648
RTD 0–100 msec (Nm sec^−1^) 4s	1270 ± 276	1340 ± 315	1376 ± 326	1276 ± 335
RTD 0–200 (Nm sec^−1^) 4sec	1799 ± 381	1852 ± 405	1947 ± 384	1849 ± 409
RTD 0–300 (Nm sec^−1^) 4 sec	2015 ± 386	2027 ± 483	2080 ± 412	2031 ± 447

RTD, rate of torque development.

**Table 4:** Concentric power and velocity produced for Constant Load (CL) and Eccentric Overload (EO) immediately following 2 and 4s eccentric contractions

	CL		EO	
	2 sec	4 sec	2 sec	4 sec
Power (W)	212.6 ± 101.2	194.2 ± 89	216.7 ± 110.5	174.5 ± 86.7
Velocity (rad.sec^−1^)	266.6 ± 37.6	264.3 ± 46.2	269.3 ± 38.1	254.5 ± 47.2

Accuracy levels during the plateau phase of the isometric trapezoid efforts were typically >92.5%. Interday absolute and relative reliability of MUFR showed acceptable values for preintervention measurements from the 16‐19‐sec (time phase 3) phase of the contraction with a coefficient of variation in 7.1% and intraclass correlation of 0.82. Given the greater absolute reliability of time phase 3, this period of the isometric trapezoid effort alone was taken for MUFR analysis. The achieved force during this period remained constant with no significant changes observed from pre to postintervention.

## Discussion

This study demonstrated reduced firing rates of high‐threshold, but not low‐ or moderate‐ threshold motor units following EO‐2s, despite unchanged MVC. In comparison, following CL‐2, CL‐4, and EO‐4, the firing rate was maintained for all motor units. Whereas, MVC and RTD were unchanged following all different types of contractions. This suggests that EO fatigued high threshold motor units despite maintenance of MVC.

The firing rate of high‐threshold motor units declined following EO demonstrating reduced neural input into the muscle even though MVC was maintained following the exercise. Although there was no alteration in linear regression of the relationship between average MUFR and recruitment threshold, the increase in just high‐threshold and not *all* motor units was insufficient to significantly increase the overall relationship. Recordings of motor units were made during the submaximal trapezoid contraction held at 70% of MVC; therefore, the reduced firing rate of high‐threshold motor units suggests that compensation occurred to sustain the force output. This compensation could have been from increased activity of other synergistic less fatigued quadriceps muscle (Akima et al. 2002), as part of a centrally derived recruitment strategy to sustain force when VL contraction is impaired. This is further supported by work on the planter flexors which demonstrated different extents of synergistic compensation over various types of exercises (Ball and Scurr 2015). To establish this, further study is needed to measure the other quadriceps muscles following EO such as the rectus femoris and vastus medialis. Nevertheless, this effect likely explains why MVC was maintained after exercise despite reduced firing rate of high‐threshold motor units.

The cause of MUFR decline is likely to be from the 120% of 3RM load used in the eccentric phase of EO‐2s whereupon increased proprioceptive feedback (Enoka 1996; Guilhem et al. 2010; Baroni et al. 2013) from afferent neurons such as type Ib from the golgi tendon organ (tension) and Ia and II from neuromuscular spindles (length) caused the increased RMS activity we demonstrated during the dynamic contractions. However, this increased RMS activity was shown for both EO contractions but the residual decline in MUFR of high threshold motor units was only shown following the faster 2‐sec contractions, suggesting that altered neural strategy had occurred following these contractions only. This could be from tension‐regulatory mechanisms whereupon Golgi tendon organs directly depress motor neuron responsiveness. However, it has been proposed that the observed decline in MUFR rather emanate from supraspinal or spinal constraints that is altered CNS strategy (for review see Duchateau and Enoka 2016). Alternatively, it may be from greater work performed in EO versus CL, as a result of fatigue following the faster and heavier eccentric contractions. However, if so we would also expect to see differences between EO‐2 and EO‐4, which we do not, suggesting that fatigue did not exist as supported by unchanged after MVC. Furthermore, as there is an inverse relationship between load and velocity during eccentric contractions (Scott and Guillermo 2013), but a positive relationship during concentric contraction we would expect any residual neural fatigue to be greater following the slow contractions; as the load was the same for both EO velocities (120% 1RM) the demand on the muscle would have been greater during the slow contraction of the eccentric phase. Nevertheless, faster velocity eccentric training results in superior strength gain (Paddon‐Jones et al. 2001; Farthing and Chilibeck 2003) suggesting that greater stimulus is received during faster eccentric contractions. As we found no differences (2‐sec vs. 4‐sec) in the heightened RMS activity *during* the eccentric phase of the EO contractions it is possible that increased frequency of proprioception did not immediately alter gross neuromuscular recruitment but transiently reduced high‐threshold motor unit firing shortly after.

Later recruited motor units become selectively activated during eccentric exercise (Nardone et al. 1989; Howell et al. 1995; Linnamo et al. 2003) and to confirm our hypothesis we showed that later recruited motor units (i.e., higher threshold) were impaired following EO. However, it has also been reported that no such selectivity exits in studies measuring MUFR during static lengthening contractions at ≤20% MVC (Garland et al. 1996; Søgaard et al. 1996; Laidlaw et al. 2000; Stotz and Bawa 2001). Nevertheless, these are relatively low contraction intensities that would probably not have recruited all high‐threshold motor units at maximum MUFR. This is unlike our study that measured the residual effect of dynamic lengthening contractions (85 and 120% of 1RM) at 70% MVC which would have placed much greater demands on involved skeletal muscle. Motor unit control during lengthening contractions is largely task specific (Duchateau and Enoka 2016) and we used contraction intensities similar to those used for EO training (Walker et al. 2016). As such, in our study, it is likely that frequent activation of high threshold motor units from the fast EO contractions reduced MUFR. This is an important finding that advances our understanding of motor unit behavior in response to EO. Furthermore, higher threshold motor units associate with type II muscle fibers (Henneman 1985) and it is these fibers that hypertrophy to contribute to strength gain following eccentric training (Paddon‐Jones et al. 2001). As such, this suggests that during fast CL contractions the eccentric phase is undertrained and to use EO instead will offer superior neural stimulus for adaptation; this has important implications for practitioners and researchers working from athletic to clinical populations.

## Conclusion

This is the first study to demonstrate an acute transient decline in high threshold motor unit firing following eccentric overload without affecting maximal force capacity. This may be a direct response to increased lengthening contractions from higher force production at fast velocity. This provides insight into part of the physiological stimulus responsible for enhancing training adaptations to eccentric overload, previously reported. This insight justifies further study, using advancing EMG decomposition technology, to measure motor unit behavior during as opposed to after eccentric overload contractions.

## Conflict of Interest

None of the authors have any conflicts of interest to declare.
